# Rheological Characteristics of Fe–C–Cr(Ni) Alloys

**DOI:** 10.3390/ma16072656

**Published:** 2023-03-27

**Authors:** Silvie Rosypalová, Lenka Řeháčková, Vlastimil Novák, Monika Kawuloková, Petra Váňová, Kateřina Konečná, Barbora Ďuricová

**Affiliations:** Faculty of Materials Science and Technology, VŠB-Technical University of Ostrava, 17. listopadu 15, Poruba, 708 00 Ostrava, Czech Republic

**Keywords:** chromium, nickel, viscosity, flow curve, viscosity curve, low-alloy steel

## Abstract

The principal objective of this project was to investigate the rheological properties of Fe–C–Cr and Fe–C–Ni-based low-alloy steels using an Anton Paar high-temperature rotational viscometer up to 1550 °C. The emphasis was placed on determining the liquidus temperatures and evaluating the flow and viscosity curves and the temperature dependence of dynamic viscosity. All were studied depending on the change in the content of chromium (0.010–4.863 wt%), nickel (0.001–4.495 wt%), and carbon (0.043–1.563 wt%). It was shown that the dynamic viscosity decreases with increasing nickel content and increases with increasing carbon and chromium content. The experimental data of the flow curves were fitted using the Herschel–Bulkley model with a good agreement between the measured and calculated values. Characterization of the internal structure was performed by SEM and EDX analyses, confirming non-significant changes in the microstructure of the original and remelted samples. The phase composition of the selected samples was also determined using JMatPro 12.0 simulation software (Sente Software Ltd., Guildford, UK).

## 1. Introduction

Since their introduction, steels have made significant progress and have ceased to be “mere” iron-carbon alloys, with improved properties such as fracture resistance and strength. Today, steels are modified with various alloying elements added, often in minute quantities, to suit specific applications. Advances in computer technology and an ever-expanding range of material-testing instruments have facilitated the production of steels with a narrow compositional scope and a wide variety of properties [1]. Low-alloy steels with compositions ranging from 2 wt% to 10 wt% of alloying elements belong to a broad group of ferrous materials with a wide range of potential uses. Significant are the ones mainly containing nickel, chromium, and other alloying elements such as vanadium, niobium, and titanium [2]. It is well-established that nickel and chromium enhance the mechanical properties of low-alloy steels, especially strength, toughness, and hardenability, which is reflected in the microstructural changes after quenching [3,4,5]. Low-alloy steels are used for nuclear pressure vessels, steam generators, and other applications in nuclear power plants where conventional steels do not meet the required endurance strength. From this perspective, they are essential materials for ensuring higher safety and durability of nuclear power plants and contribute to increasing power generation efficiency [6,7,8]. However, low-alloy steels are also used as special structural parts in the aerospace and automotive industries and in the production of gears and crankshafts, where high requirements are imposed for high tensile strength, temperature resistance, corrosion resistance, fatigue resistance, and fracture toughness [9,10,11,12,13]. Despite their widespread use, mainly due to the advantageous combination of their cost and unique properties, there is still a paucity of thermophysical and especially experimental data in the literature regarding their rheological properties.

The determination of viscosity and other rheological parameters of molten metals, steels, and alloys is highly demanded as they play a crucial role in mass transfer processes and enable the design and optimization of the melting, casting, and welding processes of Fe-based alloys [14,15]. This determination is also challenging because the systems under investigation have high liquidus temperatures, oxidize easily, and the stability of the measuring system must be ensured during experiments. In addition, the measured viscosity values are typically in the order of mPa·s [16,17].

The main component of low-alloy steels is iron, whose viscosity measured at melting temperature is about 6 mPa·s. Specifically, Chapman determined it to be approximately 6.5 mPa·s [18], Battezzati 5.5 mPa·s [19] and Brooks 6.93 mPa·s [16]. However, even in the case of binary and ternary systems, the viscosity of iron-based alloys can vary by units of mPa·s, with the addition of alloying elements in the order of tenths to units of percent [20,21]. Furthermore, it is worth noting that the effect of the same dissolving element on viscosity may differ for binary and multicomponent melts, and it can be assumed that specific interactions between the components change the patterns of their effect on viscosity [22]. Over the past two decades, studies have been performed addressing the effect of chromium and nickel on the viscosity of binary and more complex systems. Sato examined the viscosities of binary systems, including Fe–Ni, over the whole concentration range using an oscillating viscometer up to 1600 °C, showing a good match with Arrhenius linearity [23]. A similar effect of nickel on the viscosity of Fe–Ni binaries was found in article [24]. A study of the dependence of kinematic viscosity on chromium content in the Fe–Cr melts showed that the viscosity isotherm is nonmonotonic with a minimum at 5 wt% and a maximum at 12 wt%. The increase in crystallization tendency was related to atoms’ geometric and chemical arrangement over short distances [25]. The viscosities of the Cr–Fe–Ni ternary system were studied at elevated temperatures, and it was found that the viscosities increased monotonically with increasing iron and chromium content [14]. Liu calculated isoviscosity curves of the ternary Fe–Ni–Cr system using Gibbs free energy of mixing and geometrical models operating with excess activation energies of sub-binary systems. Nickel decreased viscosity over the entire concentration range, but chromium only did so at contents exceeding 20 mol% [26]. The viscosity of Fe–Cr–Mn–Ni alloys with nickel contents up to 20% in the temperature range of 1723–1873 K was measured using a vibrating finger viscometer. Nickel was found to decrease viscosity within this range, which was related to the change in the primary solidification structure from a body-centered cubic unit cell to a face-centered cubic unit cell [27]. The effect of nickel on the viscosity of Fe-based multicomponent melts was evaluated in [28], where nickel decreased the viscosity and increased the activation energy, with the change in viscosity being related to structural changes and decomposition of high-temperature clusters of cementite and silicon oxides. A large amount of published data on the viscosity of metals, alloys, and intermetallic compounds is given in [19].

The present study was designed to determine the effect of alloying elements such as nickel, chromium, and carbon on the rheological properties of selected low-alloy steels. Since investigated systems were poly-component and, in these cases, the properties are difficult to calculate or simulate through advanced applications, this study sought to obtain data that would help address corresponding research gaps. For these reasons, the measurements were performed on a highly sensitive instrument under conditions not significantly affecting the composition and structure of the specimens.

## 2. Materials and Methods

### 2.1. Sample Preparation

Alloy samples were prepared from pure metals (Fe, Ni, Cr, purity 99.99%), carbon (purity 99.99%), and Fe_2_O_3_ tablets (purity 99.999%) by vacuum induction melting using a Leybold Heraeus furnace. The melt was cast into the vertically oriented mold, yielding 3 kg ingots from which rods of diameter 27 mm and, subsequently, cylindrical specimens (27 mm diameter × 38 mm height) were made. The chemical composition of all samples, determined by a Spectruma GDA 750 HP optical emission spectrometer (GDOES), is listed in Table 1. The carbon, oxygen, and sulfur contents were determined by Eltra 200 CS and Eltra 2000 ONH combustion analyzers.

### 2.2. Determination of Liquidus Temperature

Differential thermal analysis (DTA), 3D differential scanning calorimetry (3D DSC), and an optical method were used to determine the liquidus temperature [29]. A Setaram SETSYS 18TM laboratory system and a Setaram Line 96 Multi High-Temperature Calorimeter (MHTC) were used for DTA and DSC analyses, respectively. The samples were analyzed in high-purity corundum crucibles. Before analyses, the alloys with the approximate masses of 190 mg (DTA) and 1200 mg (DCS) were brushed and cleaned in acetone. A dynamic atmosphere of Ar (purity 99.9999%) was maintained to protect the samples against oxidation. Liquidus temperatures of each alloy were obtained throughout the heating runs. The DTA and DCS runs were carried out at a heating rate of 10 °C·min^−1^ and 5 °C·min^−1^, respectively. The obtained liquidus temperatures were corrected for the melting temperatures of high-purity metals, Ni and Pd, and for the experimental conditions.

The optical method was carried out by sessile drop in a CLASIC high-temperature observation furnace. The alloy sample was placed in an Al_2_O_3_ substrate and inserted into the furnace tube, which was hermetically sealed, evacuated to 0.1 Pa, and purged with Ar (purity 99.9999%). Liquidus temperatures were determined optically based on changes in the sample silhouettes taken with a CANON EOS 550D during heating (heating rate of 5 °C·min^−1^).

### 2.3. Determination of Rheological Properties (Parameters)

The rheological measurements were carried out with an Anton Paar FRS 1600 high-temperature rotational viscometer (Anton Paar GmbH, Graz, Austria). This instrument combines a laboratory furnace and a DSR 301 measuring head with air bearings. The furnace allows measurements of up to 1550 °C registered by a Pt–13% Rh/Pt thermocouple. The rheometer is air-cooled to protect mechanical and electronic components from overheating. The measuring system consists of an alumina spindle mounted on a long ceramic shaft connected to the rheometer head and an alumina crucible fixed to a lower ceramic shaft. The experiments were conducted in rotation mode by measuring the torque of a spindle rotating in a crucible filled with molten alloy.

Prior to the experiment, the alloy samples were thoroughly cleaned mechanically to remove surface oxides. The corundum crucible containing the alloy sample was placed in the furnace. To prevent oxidation of the samples, a gas mixture of argon (99.9999% purity) and hydrogen (2.6 vol%, 99.999% purity) was used at a flow rate of 150 L·h^−1^. The furnace was heated to 1550 °C at a heating rate of 17 °C·min^−1^. The sample was kept at this temperature for 150 min for temperature stabilization and homogenization. Subsequently, the alumina spindle was immersed in the melt, and flow curves were recorded at a temperature of 1550 °C. Based on measurements of viscosity dependence on the shear rate, an optimum shear rate of 10 s^−1^ was chosen for the viscosity measurement, performed during cooling at a rate of 2.5 °C·min^−1^ in the temperature range from 1550 °C to the temperature at which the samples began to solidify.

### 2.4. SEM and EDX Methods

Firstly, metallographic samples were polished and etched (nital etching process). Consequently, the structures were examined using an Olympus IX70 (LM) light microscope (Olympus, Melville, NY, USA) and a JEOL 6490 LV scanning electron microscope ((JEOL Ltd., Akishima, Japan)) operating in a secondary electron mode, equipped with an INCA EDX (Energy Dispersive X–ray Spectroscopy) analyzer (Oxford Instruments, Oxford, UK) enabling X-ray analysis. The SEM settings were as follows: thermionic cathode LaB6, voltage 20 kV, and the specimen chamber kept at 10^−3^ and 25 Pa.

## 3. Results and Discussion

### 3.1. Liquidus Temperatures

The liquidus temperatures were obtained using three experimental methods: DTA, DSC, and optical. The experimentally obtained values were then compared with those theoretically calculated by ThermoCalc 2019a software. All temperatures are listed in Table 2.

Good agreement was observed when comparing the liquid temperatures obtained using the DTA and DSC methods, with a maximum difference not exceeding 5 °C. However, concerning the temperatures obtained by the optical method, the differences were more significant, especially for samples 3 (0.344 wt% C, 0.924 wt% Cr), 7 (0.043 wt% C, 4.863 wt% Cr), and 8 (1.378 wt% C, 4.591 wt% Cr), where the maximum difference was 13 °C for sample 8. A possible explanation for this might be that the optical method considers the temperature of the liquid as that at which the sample assumes a perfect drop shape. It is worth noting that determining liquid temperatures at high temperatures entails several challenges, including those relating to the experimental setup, experimental conditions (heating rate, sample weight), or changes in the chemical composition of the samples during heating (oxidation, decarburization) [30,31,32]. As for the values calculated with ThermoCalc 2019a software (Thermo-Calc Software, Stockholm, Sweden), one must consider certain simplifications that the software operates with, e.g., the absence of certain elements, equilibrium conditions, and others.

### 3.2. Flow and Viscosity Curves

**Figure 1:**
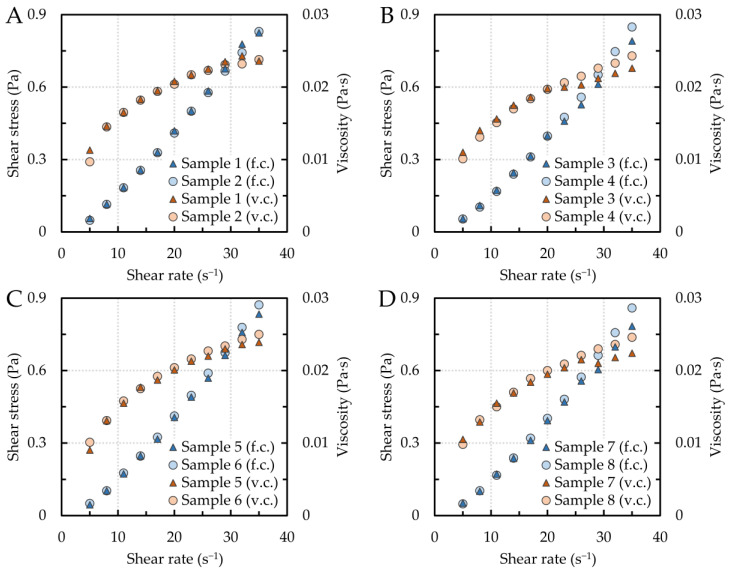
Flow (f.c.) and viscosity (v.c.) curves for samples (**A**) 1 and 2, (**B**) 3 and 4, (**C**) 5 and 6, and (**D**) 7 and 8.

The flow characteristics of systems in the liquid state respect the rheological equations of state describing the relationship between shear stress and fluid deformation. The flow behavior can be represented by the flow and viscosity curves. Based on their shape, the Newtonian or non-Newtonian behavior of the melt under investigation can be determined. For Newtonian melts, the shear stress is directly proportional to the shear rate, and the viscosity depends only on the temperature, i.e., it is independent of the shear rate. In the case of non-Newtonian melts, the viscosity is dependent on the shear rate. Figure 1 shows flow and viscosity curves for all samples at 1550 °C. The flow curves are presented as the dependence of shear stress on shear rate, and the viscosity curves as the dependence of viscosity on shear rate. All dependencies were measured in the shear rate interval of 5–35 s^−1^. For all alloys, shear stress and viscosity increased non-linearly with shear rate. From this, it can be concluded that all the alloys investigated exhibit a type of non-Newtonian behavior, i.e., shear thickening.

The experimental data of the flow curves obtained at 1550 °C were fitted with the Herschel–Bulkley model [33] according to Equation (1):

(1) $$\tau =\tau_{0}+k{\dot{\gamma }}^{n}$$

where τ (Pa) is the shear stress, $\tau_{0}$ (Pa) is the yield stress, k (Pa·s^n^) is the consistency index, $\dot{\gamma }$ [s^−1^] is the shear rate, and n [-] is the flow index.

**Figure 2:**
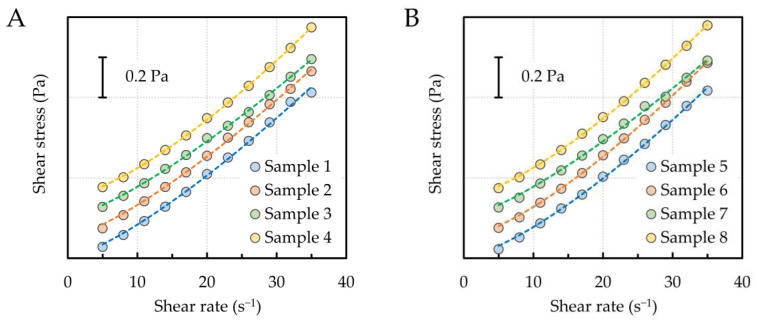
Flow curves fitted by the Herschel–Bulkley model (dashed lines denote the fitting curves); (**A**)–samples 1–4, (**B**)–samples 5–8.

Non-linear least squares analysis involving a generalized reduced-gradient optimization algorithm [34] was used to optimize the model parameters listed in Table 3. The fitting curves are shown in Figure 2. Excellent agreement was reached between the experimental and theoretical data, as evidenced by the values of the correlation coefficients and the error sum of squares (SSE).

### 3.3. Temperature Dependence of Dynamic Viscosity

**Figure 3:**
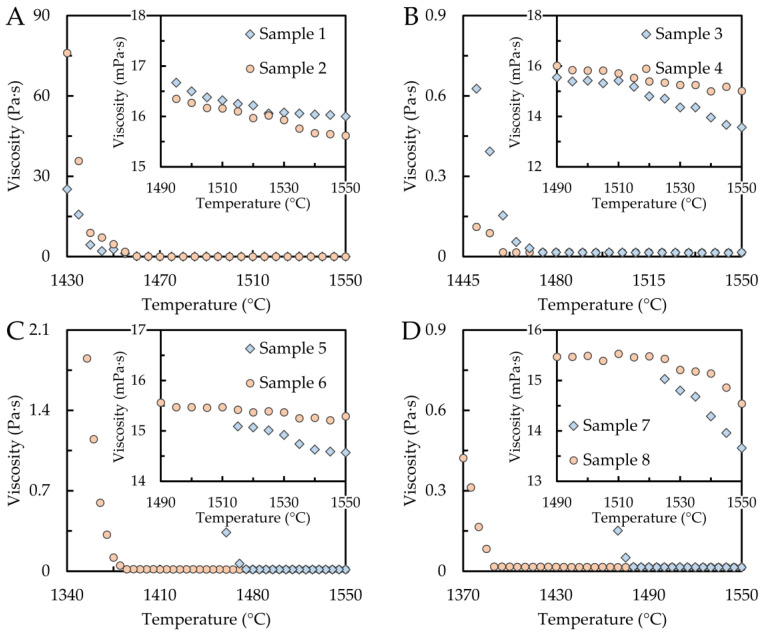
Temperature dependencies of viscosity for the samples (**A**) 1 and 2, (**B**) 3 and 4, (**C**) 5 and 6, and (**D**) 7 and 8.

The temperature dependence of the dynamic viscosity of the samples was experimentally investigated during the cooling process, i.e., in the temperature interval from the maximum temperature (1550 °C) to the solidification temperature. The obtained dependencies are shown in Figure 3A–D. As shown in the figure, the dynamic viscosity increases exponentially with decreasing temperature, which agrees with the Arrhenius equation [35]. The details show the dependence of the dynamic viscosity on temperature when the sample is in the liquid state. It can be argued that the effect of chemical composition—Ni, Cr, and C contents—is almost negligible in the investigated concentration ranges of Cr (0.924–4.796 wt%), Ni (1.084–4.478 wt%), and C (0.043–1.378 wt%). However, a slight increase in viscosity with increasing chromium content can be observed in Figure 3B, where sample 3 with 0.924 wt% chromium had a viscosity of 13.6 mPa at 1550 °C, while sample 4 with 4.796 wt% chromium had a viscosity of 15.0 mPa at the same temperature. It is worth noting that a similar effect of chromium was observed for ternary alloys containing chromium and nickel, but the chromium content varied in the order of tens of percent [14,26]. A similar trend can be observed for increasing carbon content (Figure 3C,D). For samples 5 (0.043 wt% C) and 6 (1.563 wt% C), containing roughly the same nickel content of about 4.5 wt%, the dynamic viscosity values at the maximum temperature were 14.7 and 14.6 mPa, respectively. Additionally, for samples 7 (0.043 wt% C) and 8 (1.378 wt% C) with approximately the same chromium content, the viscosity increased slightly from a value of 13.4 mPa to a value of 14.5 mPa. A slight decrease in viscosity can be observed with increasing nickel content (Figure 3A), yielding viscosities of 16.0 mPa for sample 1 (1.084 wt% Ni) and 15.2 mPa for sample 2 (4.478 wt% Ni). In the same vein, Dubberstein described a moderate decrease in viscosity depending on the nickel content for Fe–Cr–Mn–Ni alloys with 3–6 wt% Ni [27].

### 3.4. Results of SEM and EDX Analyses

**Figure 4:**
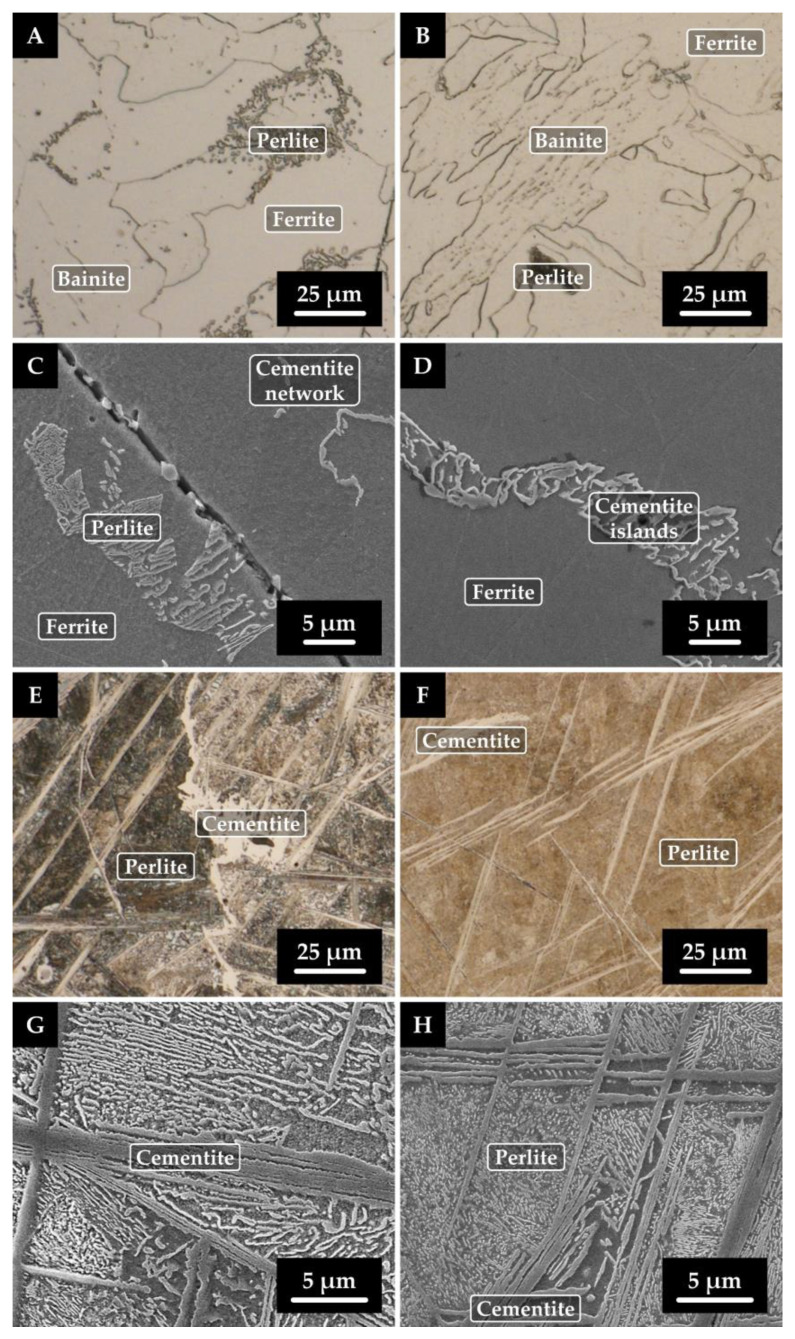
Microstructure of nickel sample 5 (**A**–**D**) with lower carbon content and sample 6 (**E**–**H**) with higher carbon content; left column—initial state, right column—remelted state; (**A**,**B**,**E**,**F**)—light microscopy, (**C**,**D**,**G**,**H**)–scanning electron microscopy.

**Figure 5:**
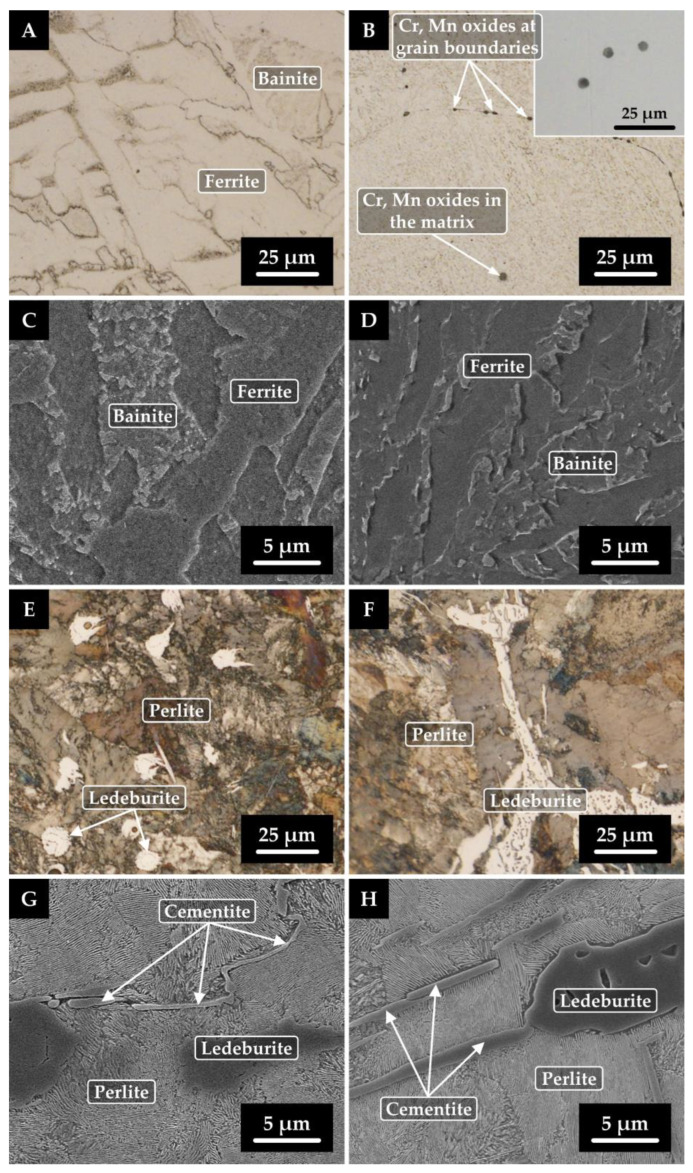
Microstructure of chromium sample 7 (**A**–**D**) with lower carbon content and sample 8 (**E**–**H**) with higher carbon content; left column—initial state, right column—remelted state; (**A**,**B**,**E**,**F**)—light microscopy, (**C**,**D**,**G**,**H**)—scanning electron microscopy. Locations where EDX spot microanalysis was performed (inlay of Figure 6B).

**Figure 6:**
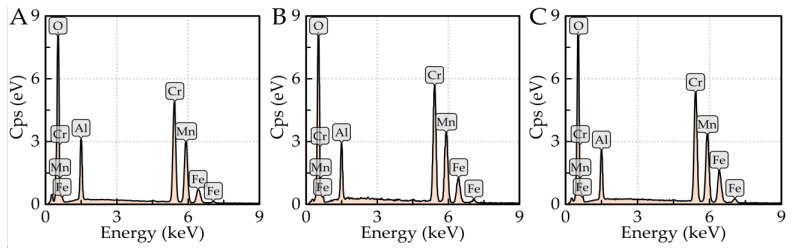
EDX spectra collected from spot microanalysis of sample 7; (**A**–**C**) correspond to spectra 1, 2, and 3.

Samples with significantly varying carbon contents and the maximum amount of alloying element (Cr and Ni) were tested using SEM and EDX analyses in both the initial state and after rheological experiments (after high-temperature testing). Specifically, samples 5 (0.043 wt% C; 4.465 wt% Ni), 6 (1.563 wt% C; 4.495 wt% Ni), 7 (0.043 wt% C; 4.863 wt% Cr), and 8 (1.378 wt% C; 4.591 wt% Cr) were investigated for changes in internal structure. The results of these analyses are shown in Figure 4 and Figure 5. Figure 4A–D and Figure 6A–D show the microstructures of the samples with low carbon content, i.e., samples 5 and 7 (0.043 wt% C). When comparing the microstructures of these samples in the initial and remelted states, it can be surmised that no significant changes in their internal structure occurred during the rheological measurements. In both samples, bainitic ferrite or bainite is present in the initial and remelted states. Sample 5 (4.465 wt% Ni) contains minor amounts of perlite and a coarse cementite network. For sample 7 (4.863 wt% Cr), bainitic ferrite and bainite are more etchable. In the center of this sample in the remelted state, complex oxide inclusions (Cr or Mn oxides) were detected in the bulk grain and along the grain boundaries (Figure 5B). This was supported by EDX analysis, the results of which are shown in Table 4 and Figure 6, containing the most representative spectra of EDX spot microanalysis. The presence of these oxides is due to the order of magnitude higher oxygen content of this sample compared to the other specimens. Figure 4E–H and Figure 5E–H show the microstructure of samples 6 and 8 with a higher carbon content (1.563 and 1.378 wt% C). In both cases, a dominant structure of lamellar perlite is observed. For sample 6 (4.495 wt% Ni), cementite plates are present in the initial state, including a fine cementite network excluded along grain boundaries. However, after rheological testing, only plates of cementite are present. The lamellae of pearlite appear finer after the rheological experiment. In sample 8 (4.591 wt% Cr), globular islands of ledeburite are present in the initial state and are altered in the remelted state to larger blocks of reticulated ledeburite along grain boundaries.

**Figure 7:**
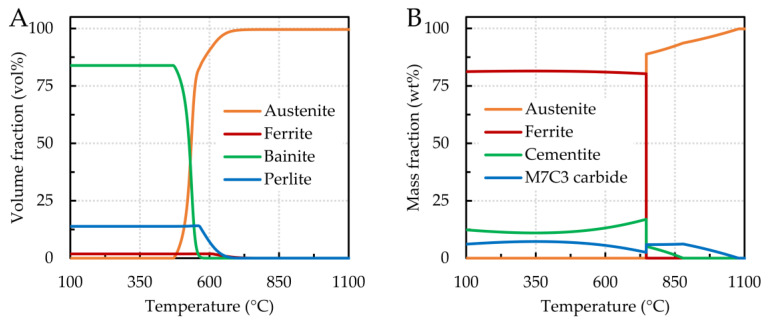
Phase formation as a function of temperature—(**A**) sample 7, 0.043 wt% C, 4.863 wt% Cr; (**B**) sample 8, 1.378 wt% C, 4.591 wt% Cr.

The results of the SEM/EDX analyses were supplemented by modeling in JMatPro simulation software, which calculates a wide range of alloy properties and focuses on multicomponent alloys of significant industrial importance. We targeted the phase composition of samples 7 (0.043 wt% C, 4.863 wt% Cr) and 8 (1.378 wt% C, 4.591 wt% Cr), and the results of the calculations are shown in Figure 7. For sample 7, nonequilibrium structural phases were assumed due to the presence of bainite. Based on the simulated phases of sample 7 (Figure 7A), bainite is the dominant phase. However, ferrite was also present in the structure of the samples considered for the experiment. The calculated equilibrium structural phases of sample 8 are shown in Figure 7B. In this sample, the presence of perlite, a lamellar mixture of ferrite and cementite, was confirmed. As mentioned above, the presence of not only perlite but also ledeburite, a mixture of cementite and perlite, was confirmed for the real sample. In the theoretical calculation, ledeburite was not detected in the structure, and the sample’s composition does not correspond to the region of ledeburite formation in the iron-cementite phase diagram, where ledeburite occurs in alloys with carbon contents higher than 2.11 wt% [36]. However, carbide-forming elements such as Cr and Mn also promote the formation of ledeburite. Sample 8 contains 1.378 wt% C but also 4.591 wt% Cr and 0.047 wt% Mn. It can be assumed that the larger amount of chromium probably caused the displacement of the ledeburite formation. The differences between the calculated compositions and those obtained by SEM analysis could be because the calculations use simplifications that do not fully reflect the actual processes occurring in the sample throughout the experiment.

## 4. Conclusions

The results of this experimental study on the rheological properties of Fe–C–Cr- and Fe–C–Ni-based low-alloy steels can be summarized as follows:

- The liquidus temperature of the alloys studied decreased with increasing carbon content in the samples. The highest liquidus temperature was detected for sample 7, with the lowest carbon content and the highest chromium content (0.043 wt% C, 4.495 wt% Cr), and the lowest liquidus temperature was found for sample 6, with the highest carbon and nickel contents (1.563 wt% C, 4.495 wt% Ni).
- The flow properties of the alloys studied were represented by flow and viscosity curves. Based on their shape, it was found that all samples exhibited non-Newtonian behavior since the shear stress increased non-linearly with the shear rate, as in the case of dynamic viscosity.
- All samples under study showed an exponential increase in viscosity with decreasing temperature. The effect of chromium, nickel, and carbon on the dynamic viscosity value in a given concentration range was minimal. A slight increase in viscosity was observed with the addition of chromium and carbon, while the viscosity decreased moderately with the addition of nickel.
- Changes in the microstructure of the selected samples were examined using SEM and EDX analyses, and it was found that no significant changes in the internal structure occurred during high-temperature rheological testing.

The determination of rheological properties, especially the viscosity of metallic melts, is essential, for example, in casting, because viscosity controls the transport rate of liquid metals, which is intimately related to the formation of casting defects such as hot tearing and increased porosity.

## Figures and Tables

**Table 1:** Chemical composition of Fe–alloys (wt%).

Sample	C	Cr	Ni	O	P	S	Mn	Cu	N	Ti	W
1	0.382	0.010	1.084	0.002	0.004	0.006	0.030	0.014	0.003	0.004	<0.001
2	0.338	0.010	4.478	0.001	0.005	0.006	0.031	0.012	0.003	0.003	<0.001
3	0.344	0.924	0.001	0.002	0.005	0.068	0.056	0.007	0.026	-	-
4	0.34	4.796	0.001	0.002	0.002	0.006	0.042	0.005	0.001	0.010	0.044
5	0.043	0.013	4.465	0.005	0.004	0.006	0.062	0.007	0.002	0.003	<0.001
6	1.563	0.011	4.495	0.002	0.005	0.006	0.046	0.009	0.003	0.004	<0.001
7	0.043	4.863	0.001	0.022	0.004	0.064	0.053	0.006	0.024	-	-
8	1.378	4.591	<0.001	0.011	0.004	0.054	0.047	0.007	0.016	<0.001	0.038

**Table 2:** Measured and calculated liquidus temperatures (°C).

Sample	DTA	DSC	Optical Method	ThermoCalc
1	1495	1498	1502	1503
2	1492	1493	1500	1497
3	1501	1504	1512	1506
4	1496	1500	1496	1501
5	1514	1515	1516	1516
6	1405	1406	1404	1403
7	1522	1527	1532	1524
8	1417	1421	1408	1428

Elements that were not included in the equilibrium calculations were: P, O, Cu, N, Ti, and W.

**Table 3:** Optimized parameters of the Herschel–Bulkley model.

Sample	10^3^ $\tau_{0}$ (Pa)	10^3^ k (Pa·s^n^)	n	R^2^	SSE
1	1.2	8.2	1.3	0.9989	0.0017
2	1.9	8.1	1.3	0.9994	0.0009
3	1.1	7.8	1.3	0.9995	0.0006
4	1.5	5.9	1.4	0.9999	0.0001
5	1.3	6.7	1.4	0.9994	0.0009
6	0.9	6.3	1.4	0.9998	0.0004
7	1.7	7.7	1.3	0.9989	0.0014
8	1.4	5.9	1.4	0.9998	0.0003

**Table 4:** EDX point analysis of oxide inclusions of sample 7.

Spectrum	O	Al	Cr	Mn	Fe
	(wt%)				
1	29.2	6.6	31.5	20.3	12.6
2	29.7	7.3	32.6	21.0	9.4
3	31.9	8.9	32.6	21.3	5.3

## Data Availability

The data presented in this study are available on request from the corresponding author.

## Abbreviations

DTA	Differential Thermal Analysis
EDX	Energy Dispersive X-ray Spectroscopy
GDOES	Glow Discharge Optical Emission Spectrometry
SEM	Scanning Electron Microscopy
